# The interrelationship among physical activity, smartphone addiction, loneliness, and sport motivation in Chinese college students: a cross-sectional network analysis

**DOI:** 10.3389/fpsyg.2026.1720457

**Published:** 2026-06-25

**Authors:** Wenzhi Hou, Haijing Song, Wanli Deng, Lin Zhou

**Affiliations:** 1 Physical Education College, Minzu Normal University of Xingyi, Xingyi, China; 2 Section of Psychology, Guizhou Vocational and Technical College of Water Resources and Hydropower, Guiyang, China; 3 School of Art and Design, East University of Heilongjiang, Harbin, China

**Keywords:** cross-sectional network analysis, loneliness, physical activity, smartphone addiction, sport motivation

## Abstract

**Background:**

The rapid digital transformation in China has posed significant challenges to the lifestyles of youth, most notably characterized by physical inactivity and heightened mental health risks among university students. Clarifying the systemic interaction mechanisms between smartphone addiction, loneliness, and sport motivation is essential for developing effective behavioral intervention frameworks in the digital age.

**Objective:**

Drawing on Psychological Network Analysis (PNA), this study explores the topological interaction structures and cross-domain association pathways among physical activity, smartphone addiction, loneliness, and sport motivation in Chinese university students.

**Methods:**

A cross-sectional design was employed, involving a large-scale sample of 10,676 full-time Chinese university students. A 15-dimensional psychological network was constructed using a LASSO-regularized Gaussian Graphical Model (GGM). Centrality metrics were utilized to identify core hubs, while bridge centrality was applied to detect cross-domain spillover effects. Additionally, the Network Comparison Test (NCT) was conducted to evaluate topological robustness and heterogeneity across genders.

**Results:**

The network topology revealed that Stimulation Orientation and Achievement Orientation within the sport motivation domain occupied the system’s core, demonstrating high strength centrality. Regarding cross-system associations, Amotivation yielded highest in both bridge strength and two-step expected influence, serving as the pivotal functional link between the sport motivation cluster and the smartphone addiction community. Loneliness emerged as another critical bridge node, significantly associated with both addictive symptoms and the depletion of autonomous motivation. Local edge analysis indicated a significant negative correlation between smartphone-related loss of control and exercise duration. While global network strength was consistent across genders (*p* = 0.456), significant topological heterogeneity was observed (*p* < 0.001), reflecting distinct gender-specific association patterns in “emotional escape” and “behavioral persistence” pathways.

**Conclusion:**

This study elucidates the central role of sport motivation and the cross-domain hub function of amotivation, reflecting an integrated framework of Self-Determination Theory (SDT) and the I-PACE model. The identified key topological sites provide systemic evidence for developing targeted, gender-sensitive intervention strategies that focus on “bridge variables” within the Chinese cultural context.

## 1 Introduction

The rapid advancement of high technology has profoundly transformed the lifestyles and mental health of university students. While the ubiquity of smartphones enhances the convenience of information acquisition, it also correlates with reduced social interaction and exacerbated negative psychological outcomes, such as heightened loneliness and diminished attention spans (Sorokowski and Sobczak, 2025). Smartphone addiction is closely associated with various mental health risks; specifically, higher levels of addictive smartphone use co-occur with diminished self-regulatory capacity, which is associated with lower participation in physical activity and weakened social motivation (Ye et al., 2025; Aldbyani et al., 2025). Furthermore, excessive smartphone use is associated with the reinforcement of loneliness as an emotional precursor, which often corresponds to a loss of interest in collective offline activities (Zhu et al., 2025; Gong et al., 2023).

Within the specific cultural context of China, these associations exhibit unique characteristics. Influenced by collectivist values and campus management models, smartphones have evolved beyond mere functional tools to become essential media for social integration. Concurrently, physical activity carries significant weight as a social norm. Therefore, exploring the interaction mechanisms within this specific psychological network is vital for developing culturally sensitive intervention strategies.

The underlying logic of these complex relationships can be elucidated through the lens of motivation and emotional regulation. According to Self-Determination Theory (SDT), autonomous motivations, such as achievement orientation, constitute the cornerstone of healthy behaviors. Meanwhile, the Interaction of Person-Affect-Cognition-Execution (I-PACE) model suggests that individuals experiencing negative emotions like loneliness often seek compensatory gratification via smartphones. This compensatory behavior is associated with smartphone addiction, reflecting an antagonistic relationship with real-world physical exercise. Crucially, pathological dependence relates to a “motivational vacuum,” leading to a state of amotivation. Within a psychological network, this state may function as a bridge connecting negative addiction systems with positive physical activity systems, potentially sustaining a self-reinforcing negative cycle.

Because traditional linear modeling often fails to capture non-linear systemic interactions between variables, the present study employs Psychological Network Analysis (PNA). PNA enables the identification of core and bridge nodes within the network topology, allowing for the precise detection of intervention targets. To date, no comprehensive research has integrated physical activity, smartphone addiction, loneliness, and sport motivation into a unified network framework among Chinese university students. In particular, empirical explorations merging SDT and the I-PACE model remain scarce (Lin et al., 2022; Shi et al., 2025).

To address these theoretical and empirical gaps, this study utilizes a large-scale sample of *N* = 10,676 Chinese university students. Applying LASSO-regularized network analysis, we explore the interactive structure among these variables (Wang et al., 2024). The innovations of this research are as follows: it systematically reveals the central regulatory role of sport motivation within a “behavior-emotion-addiction” framework; it identifies key cross-system bridge variables; and it provides an in-depth interpretation by integrating China’s unique generational traits (such as the only-child background) and campus cultural norms, thereby offering cross-cultural evidence for mental health interventions in non-Western contexts.

## 2 Literature review

### 2.1 Physical activity, sport motivation, and psycho-behavioral pathways

Physical activity is a key factor in promoting the physical and mental health of university students, as it relates to enhanced emotional regulation, cognitive function, self-control, and social adaptability (Yang et al., 2025). According to Self-Determination Theory (SDT), an individual’s physical activity is associated with both intrinsic motivation (e.g., interest, sense of achievement) and extrinsic motivation (e.g., social evaluation, rewards). Specifically, individuals with higher autonomous motivation demonstrate significant advantages in maintaining the stability of exercise behavior (Bourque et al., 2025; Yu et al., 2024).

From a cross-cultural perspective, recent research by Chinese scholars indicates that exercise adherence stems not only from motivational factors but is also deeply rooted in the pursuit of life meaning, while being associated with the mediating roles of psychological resilience and cognitive reappraisal (Wang and Niu, 2025). In short, the mechanism of physical activity has evolved from a single motivation-driven model into a complex system involving cognitive regulation and existential psychological resources.

Recent literature increasingly emphasizes the pivotal role of sport motivation in connecting psychological variables with healthy behaviors. Hsia et al. (2025) note that while intrinsic motivation relates to the direct promotion of physical activity, it also corresponds to the indirect alleviation of loneliness and anxiety. Furthermore, evidence from Alajlan et al. (2024) suggests that sport motivation can effectively counteract the negative association between psychological stress and exercise frequency, highlighting its central position in regulatory pathways. Despite these findings, the bridging mechanism of sport motivation within multivariate interaction systems—especially complex networks integrating addiction symptoms and emotional variables like loneliness—requires further empirical exploration.

### 2.2 Smartphone addiction and loneliness: a destructive interaction mechanism

Smartphone addiction, also referred to as problematic smartphone use (PSU), is characterized by compulsive use, emotional escape, and functional impairment, and is showing an increasing trend among university students (Ramón-Arbués et al., 2025). According to the Interaction of Person-Affect-Cognition-Execution (I-PACE) model, addictive behavior involves a long-term imbalance between individual traits, emotional regulation, and executive function (Brand et al., 2019).

Empirical studies indicate that PSU is associated with weakened self-regulation, which relates to decreased participation in physical activity (Zeng et al., 2022; D'Cruz et al., 2024). Research on college students indicated that high-intensity physical activity is associated with reduced symptoms and psychological distress related to nomophobia (the fear of losing mobile phones). This finding further reinforces the negative correlation between physical exercise and problematic mobile phone use (Rebhi et al., 2023). Loneliness often co-occurs with PSU, exhibiting a bidirectional reinforcement relationship (Xiao et al., 2026; Zhang et al., 2024). Loneliness co-occurs with a diminished sense of psychological belonging and social motivation, which is associated with lower levels of sustained physical activity (Xu and Tang, 2024). Furthermore, literature confirms that loneliness functions as a bridge variable in the “addiction-motivation-behavior” pathway; it is associated with exacerbated smartphone addiction while relating to interference in the formation of health-related motivations (García-Ortiz et al., 2025; Wang et al., 2024). This reinforcement cycle is more complex in specific socio-cultural contexts. For instance, among Chinese university students, smartphone addiction corresponds to a decline in self-concept clarity through the chained mediation of social anxiety and social withdrawal (Wang and Niu, 2026). In summary, smartphone addiction is not merely a single behavioral deviation but a comprehensive manifestation of psychosocial maladaptation, where negative emotions such as loneliness serve as both correlates of addiction and reflections of functional impairment.

### 2.3 Psychological network analysis: identifying bridge structures

While existing literature documents pairwise associations among core variables, traditional analytical methods, such as Structural Equation Modeling (SEM) and path regression, rely heavily on unidirectional causal assumptions. Consequently, these approaches often struggle to capture the complex bidirectional or non-linear interactions between variables (Epskamp et al., 2018; Hevey, 2018). In contrast, Psychological Network Analysis (PNA), rooted in graph theory, identifies core nodes and bridge pathways within a system by modeling partial correlations. This provides a robust tool for analyzing high-dimensional interactive structures.

Currently, the application of PNA in the field of exercise psychology is in its nascent stages (Hevey, 2018; Broda et al., 2023). For instance, Van Borkulo et al. (2023) note that identified regulation functions as a bridge between the psychological traits and exercise behaviors of university students. However, their model does not incorporate addiction or emotional variables, and the sample is restricted to Western cultures, which involves limitations regarding the generalizability of the findings (Cramer et al., 2010). Within the Chinese context, network-based research that systematically integrates sport motivation, smartphone addiction, loneliness, and physical activity remains scarce. The present study seeks to address this empirical gap by merging Self-Determination Theory (SDT) and the I-PACE model within a network analysis framework to reveal the deep topological relationships among these variables.

In summary, although binary links between addiction, motivation, and behavior are relatively well-established, the field lacks an integrated perspective to elucidate the “systemic effects” associated with emotional variables such as loneliness. By addressing the high-dimensional interactive issues of health behaviors within China’s digital landscape in the post-pandemic era, this study corresponds to the provision of theoretical support and practical guidance for the design of precise, multi-component interventions.

### 2.4 Theoretical integration and the present study

The integration of the Interaction of Person-Affect-Cognition-Execution (I-PACE) model and Self-Determination Theory (SDT) provides a systematic theoretical framework for elucidating the mechanisms underlying health behavior gains and losses among university students. Specifically, the I-PACE model clarifies the “affect-addiction” pathway, wherein loneliness, serving as a negative emotional context, is associated with compensatory smartphone use that involves a transition into addictive behavior (Lu et al., 2026). Conversely, SDT explains the “motivation-behavior” transformation pathway: autonomous motivation, as the endogenous driver for sustaining physical activity, corresponds to the efficacy of translating exercise intention into actual behavior.

**Figure 1:**
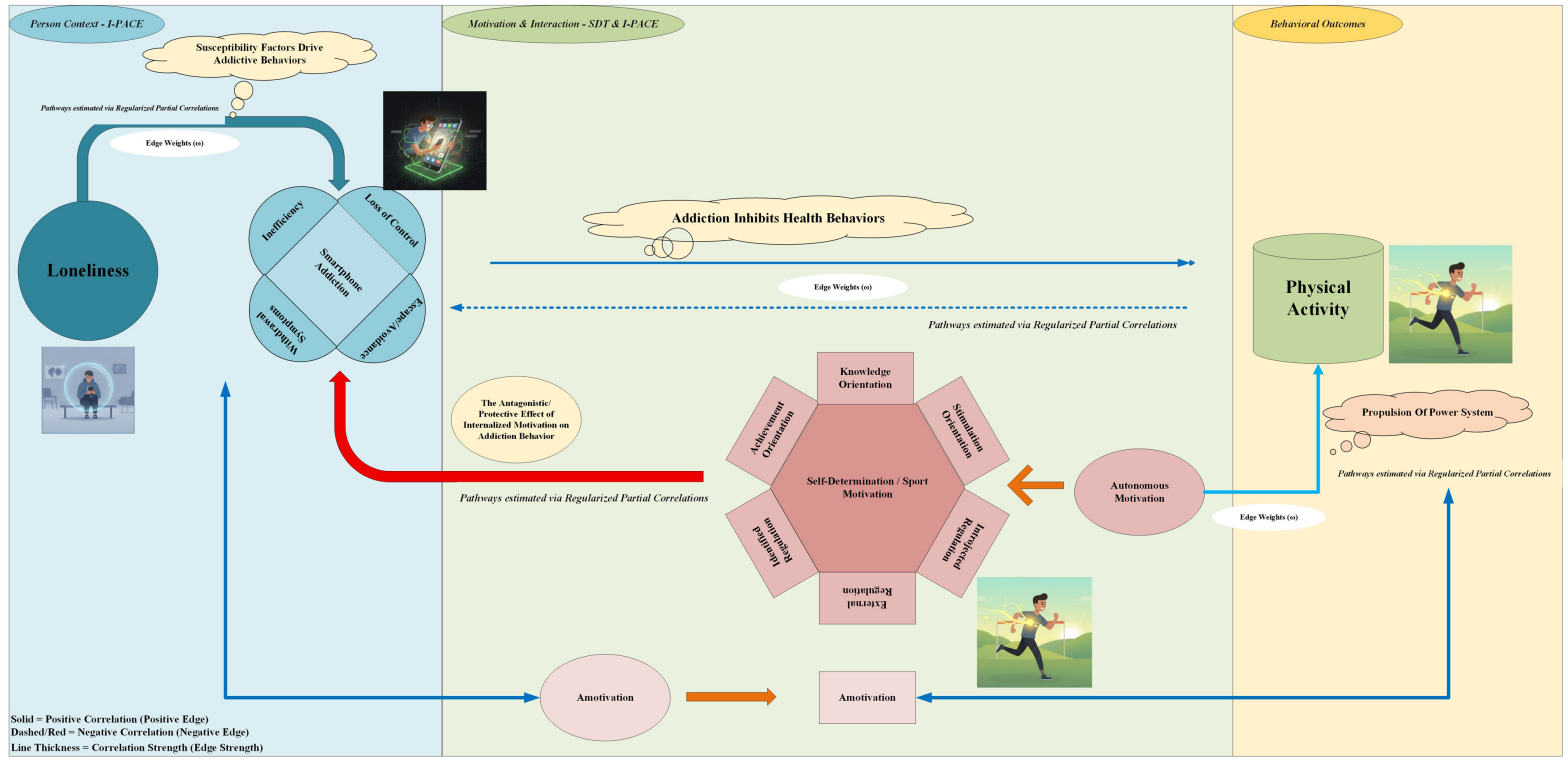
Integrated theoretical framework of the study based on the I-PACE model and self-determination theory (SDT) via psychological network perspective. Note: The blue area represents the I-PACE components (loneliness as person context; smartphone addiction as execution). The green area represents SDT components (autonomous motivation vs. amotivation). The connecting lines (edges) illustrate how Psychological Network Analysis (PNA) quantifies the dynamic associations within this integrated system.

Through this integration, the study suggests that smartphone addiction is associated with the depletion of psychological resources, which co-occurs with a diminished state of an individual’s basic psychological needs. This process corresponds to the impairment of autonomous motivation and the emergence of a “motivational vacuum” within the SDT framework. This “motivational vacuum,” facilitated by the bridging role of the “Amotivation” node, relates to the transmission of negative associations from smartphone addiction to the physical activity system, which is associated with decreased participation in physical exercise. In summary, this theoretical synthesis not only aids in identifying key topological sites of risk transmission but also reveals a systemic logic regarding the gains and losses of health behaviors among university students in the digital age (Figure 1).

### 2.5 Research hypotheses

Based on the integrated framework of SDT and the I-PACE model, as well as prior literature and the preliminary analysis of network relationships among variables, the following hypotheses are proposed:

*H1*: (SDT-Oriented) Autonomous sport motivation (e.g., stimulation and achievement orientations) represents the core functional cluster with the highest strength centrality, positively facilitating physical activity through strong positive edge weights.

*H2*: (I-PACE-Oriented) Loneliness, acting as a person-context factor, is indirectly and negatively associated with the sport motivation system via the topological pathways of smartphone addiction (e.g., loss of control and escape).

*H3*: (Integrated Hub) Amotivation (EM_Amot) serves as a cross-system bridge hub with the highest bridge strength, representing a pivotal topological link between the smartphone addiction cluster (I-PACE component) and the physical activity cluster (SDT pathway).

## 3 Methods

### 3.1 Research object and sampling strategy

#### 3.1.1 Research design and sampling

This study adopted a cross-sectional survey design to explore the potential interaction mechanisms among physical activity, smartphone addiction, perceived loneliness, and sport motivation in Chinese university students. Based on Psychological Network Analysis (PNA), a variable structure graph was constructed to identify core nodes and bridge pathways within the system.

The target population consisted of full-time undergraduate students in mainland China. Data collection was performed via the “Wenfengxing” (SurveyStar) online platform, utilizing a cluster random sampling method. The recruitment process involved university social networks, new media platforms, and student associations.

#### 3.1.2 Participants and data cleaning

A total of 11,676 questionnaires were initially collected in this study. To ensure the reliability and validity of the data analysis, the research team implemented a rigorous data cleaning procedure. Samples characterized by significant logical inconsistencies, patterned responses (e.g., selecting the same option for all items), or missing key variables were excluded. Furthermore, based on the minimum completion time threshold established during the pilot study, questionnaires completed in less than 450 s were removed to identify and eliminate cases of careless responding. After these screening processes, 1,000 invalid questionnaires were excluded, resulting in a final sample of 10,676 valid responses, with an effective recovery rate of 91.44%. This substantial sample size not only demonstrates excellent population representation but also far exceeds the requirements for parameter estimation stability in Psychological Network Analysis (PNA). This ensures the reliability of the topological structure and the robustness of the population representation through a balanced sample distribution. The detailed demographic characteristics of the participants are presented in Table 1.

**Table 1:** Demographic characteristics of the study sample (*N* = 10,676).

Dimension	Category	Percentage	Count
Gender	Male	55.1%	5,882
	Female	44.90%	4,794
Age group	Under 19 years old	17.30%	1847
	19–20 years old	19.90%	2,125
	20–21 years old	19.90%	2,125
	21–22 years old	19.00%	2028
	Over 22 years old	21.20%	2,263
Major	Humanities	25.10%	2,680
	Science and engineering	25.00%	2,669
	Medicine	20.10%	2,146
	Arts	15.10%	1,612
	Physical education	14.70%	1,569
Residence	Urban	60.70%	6,480
	Rural	39.30%	4,196

#### 3.1.3 Ethical statement

This study strictly adhered to the ethical principles of the Declaration of Helsinki and received formal approval from the Ethics Committee of Minzu Normal University of Xingyi on March 17, 2025 (Approval No.: 2025-HS-Psych-1023). Prior to data collection, all participants signed informed consent forms through the online system, ensuring that participation was informed, anonymous, and voluntary. All participants in this study were adult university students aged 18 or older; thus, no issues regarding ethical permission for minors were involved. All collected data are protected by strict confidentiality agreements and used solely for academic research purposes.

### 3.2 Research tool

The research design is based on the Self Determination Theory (SDT) and the Interaction of Person Affect Cognition Execution Model (I-PACE Model) (Elhai et al., 2025; Brailovskaia et al., 2023), combined with the Psychological Network Analysis (PNA) method to identify nonlinear relationship structures between variables (Epskamp et al., 2018).

To ensure the precision and reliability of the constructs, the core variables in this study were assessed using standardized scales that have demonstrated superior structural validity and internal consistency in prior research. Furthermore, all instruments have undergone rigorous validation for applicability within the Chinese cultural context (see Table 1 for a detailed summary).

The psychometric properties of the four scales used in this study were systematically evaluated (see Table 2). All results were derived from the original dataset of 10,676 participants. In terms of reliability, the internal consistency of each scale was excellent, with Cronbach’s *α* coefficients ranging from 0.797 (Physical Activity Rating Scale) to 0.967 (Sport Motivation Scale), both well above the widely accepted academic threshold of 0.70. Regarding validity, the results of the Confirmatory Factor Analysis (CFA) indicated that all measurement models demonstrated good to excellent fits with the empirical data. Specifically, the Comparative Fit Index and Tucker-Lewis Index for each scale ranged from 0.908 to 0.985, satisfying the recommended criterion of being greater than 0.90. Simultaneously, the Root Mean Square Error of Approximation and the Standardized Root Mean Square Residual fell within the ideal ranges of 0.023–0.071 and 0.017–0.062, respectively (both <0.08). The aforementioned statistical evidence fully confirms that the instruments selected for this study possess exceptional reliability, validity, and structural robustness within the context of this large-scale sample, providing a solid data foundation for the subsequent Psychological Network Analysis.

**Table 2:** Overview and psychometric properties of the selected scales.

Scale	Number of terms	Cronbach’s *α*	Cite	CFI	TLI	RMSEA	SRMR
Physical Activity Rating Scale (PARS-3)	3	0.797	Zhang et al. (2022)	0.985	0.978	0.023	0.017
UCLA Loneliness Scale Version 3	20	0.846	Lim et al. (2021)	0.921	0.914	0.071	0.056
Smartphone Addiction Scale (SAS)	17	0.932	Yang et al. (2023)	0.961	0.947	0.052	0.046
Sport Motivation Scale (SMS)	28	0.967	Zhang et al. (2023)	0.915	0.908	0.068	0.062

### 3.3 Data processing and statistical analysis strategies

Data processing and analysis were conducted using R (version 4.3.1) and SPSS (version 23.0). Initially, descriptive statistics were employed to analyze the distribution characteristics of variables via SPSS 23.0, with skewness and kurtosis utilized to assess data normality. For non-normally distributed psychometric data, a non-paranormal transformation (npn) was applied to enhance the robustness of partial correlation estimations. In the data processing phase, a proactive quality control strategy was adopted, with a statistical contingency plan pre-established using Multiple Imputation by Chained Equations to address potential missing data risks. However, after implementing rigorous screening based on response duration (using a 450-s threshold) and logical consistency, a systematic audit confirmed that the final valid sample (*N* = 10,676) achieved 100% data completeness, with a 0% missing rate across all variables. Given the exceptional completeness of the dataset, the final network models were constructed directly using the full original data. This decision effectively circumvented the predictive bias potentially introduced by simulated imputation and provided a reliable empirical foundation for the precise estimation of edge weights and the topological robustness analysis of the psychological networks.

In terms of core network construction, this study utilized the qgraph and bootnet packages to build a LASSO-regularized Gaussian Graphical Model (GGM). The partial correlation network was estimated using the EBICglasso algorithm, with the hyperparameter gamma (*γ*) set to 0.5 to achieve an optimal balance between model sparsity and the identification of true associations. Prior to modeling, all raw data underwent Z-score standardization.

The study extracted multiple centrality indices to evaluate the topological properties of nodes, including Strength, Betweenness, Expected Influence, and Bridge Centrality. Bridge centrality is defined as the sum of the connection strengths between a node and nodes outside its designated community; it reflects the capacity of a node to function as a “risk spillover bridge” across different psychological domains, such as the addiction system and the physical activity system.

Furthermore, the Network Comparison Test (NCT) was employed to examine topological differences across gender subgroups. To verify the robustness of the results, a non-parametric bootstrap (1,000 iterations) was executed to determine the confidence intervals for edge weights. Finally, a case-dropping bootstrap procedure was conducted to calculate the centrality stability coefficient (CS-coefficient), verifying the stability of node rankings (Servidio et al., 2022; Zhao and Kou, 2024).

Additionally, confirmatory factor analysis (CFA) for scale validation was performed using the SPSSAU online statistical analysis platform.

**Figure 2:**
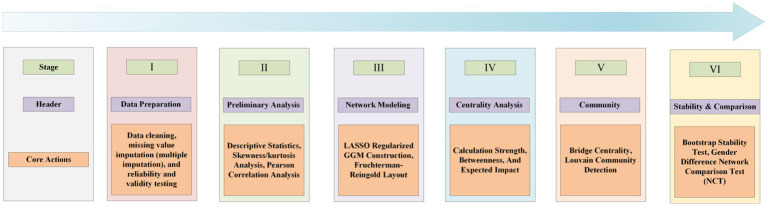
Data processing procedure. PNA, Psychological Network Analysis; GGM, Gaussian Graphical Model; LASSO, Least Absolute Shrinkage and Selection Operator; NCT, Network Comparison Test.

The specific operation process is shown in Figure 2.

The present study employed Psychological Network Analysis (PNA) rather than traditional linear modeling, such as multiple regression or Structural Equation Modeling (SEM). This choice was attributed to the inherently systemic and interactive characteristics of both Self-Determination Theory (SDT) and the I-PACE model. Traditional linear models rely heavily on unidirectional causal assumptions, which struggle to capture complex bidirectional feedback loops between variables. In contrast, based on LASSO-regularized partial correlation modeling (Epskamp and Fried, 2018), PNA presents a dynamic equilibrium among addiction, affect, motivation, and behavior within a unified topological structure.

By calculating bridge centrality (e.g., bridge strength), PNA enables the precise identification of “risk transmission pathways” in the I-PACE model and “motivation regulatory hubs” in SDT from a topological perspective. This approach translates macro-psychological theories into micro-visual architectures, allowing for a refined examination of how distal factors, such as loneliness, are associated with diminished healthy behavior patterns through specific symptom pathways, such as escape motivation. Consequently, PNA provided a robust analytical platform that aligns highly with the systemic complexity of the integrated framework used in this research.

### 3.4 Generative AI usage statement

Generative artificial intelligence (AI) tools (specifically ChatGPT-4o) were utilized in the preparation of this manuscript. The scope of AI application was strictly limited to the linguistic translation and basic grammatical refinement of the initial drafts of the Introduction and Methodology sections, both of which were originally authored in Chinese by the researchers. All other core components of the paper, including the results analysis, discussion, and conclusion, were drafted independently by the author team. To ensure linguistic consistency and the accuracy of professional terminology, the authors comprehensively reviewed and edited all AI-generated suggestions. Furthermore, academic experts and native English-speaking peers were invited to perform in-depth proofreading and logical streamlining of the entire text. AI technology was not employed for data collection, statistical analysis, the interpretation of results, or the generation of core conclusions. The authors assume full responsibility for the integrity, scientific validity, and academic rigor of the research content. This disclosure follows the recommendations for transparent reporting of AI in academic writing proposed by Dergaa et al. (2024).

## 4 Results

### 4.1 Description of subject information and correlation analysis

Table 3 presents the descriptive statistics for the 15 variables across the four primary dimensions. Regarding mean levels, the scores for physical activity intensity, duration, and frequency ranged from 2.61 to 3.35, suggesting a moderate level of physical engagement among the participants. The mean scores for the four sub-dimensions of smartphone addiction were relatively low (1.93–2.38), with “Withdrawal Symptoms” yielding the lowest value (1.9278), which indicates that pathological addiction tendencies within the sample population are not pronounced. Regarding dispersion, with the exception of the loneliness scale—which exhibited a higher standard deviation (SD = 10.46) due to its broader scoring range—the SD for all other dimensions remained between 0.70 and 1.22, reflecting the stability and consistent central tendency of the dataset. In terms of distributional characteristics, the absolute values of skewness for all variables were below 2, and the absolute values of kurtosis were below 7. These results indicate that the variables approximately followed a normal distribution, satisfying the statistical prerequisites for subsequent Psychological Network Analysis (PNA) and robust parameter estimation. Overall, this large-scale dataset provides a robust empirical foundation for elucidating the non-linear associations between adolescent mental health and physical activity behaviors.

**Table 3:** Descriptive statistics of subjects *N* = 10,676.

Dimension	Variable name	Abbreviation	Mean	SD	Skewness	Kurtosis
Physical activity	Intensity	PA_Int	2.6126	1.1822	0.1845	−0.9007
	Duration	PA_Dur	3.3497	1.2154	−0.1638	−0.9525
	Frequency	PA_Freq	3.1446	1.0744	−0.0445	−0.5177
Smartphone addiction	Loss of control	SA_LoC	2.0995	0.7499	0.7378	0.7932
	Withdrawal symptoms	SA_WS	1.9278	0.8943	1.0027	0.6312
	Escape/avoidance	SA_EA	2.3789	0.9923	0.5114	−0.262
	Inefficiency	SA_Ineff	2.2731	0.9141	0.5016	−0.0296
Sport motivation	Knowledge orientation	SMS_Know	2.6307	0.7864	−0.1529	−0.4996
	Achievement orientation	SMS_Ach	2.7249	0.8083	−0.2591	−0.4926
	Stimulation orientation	SMS_Stim	2.7184	0.807	−0.2396	−0.5159
	Introjected regulation	SMS_Ident	2.5205	0.739	−0.043	−0.314
	Identified regulation	SMS_Intro	2.5904	0.7765	−0.1038	−0.4523
	External regulation	SMS_Extern	2.4037	0.7308	0.0793	−0.324
	Amotivation	SMS_Amot	2.2141	0.706	0.2654	−0.216
Loneliness	Loneliness	Loneliness/L	46.1362	10.4599	−0.6908	0.9107

**Figure 3:**
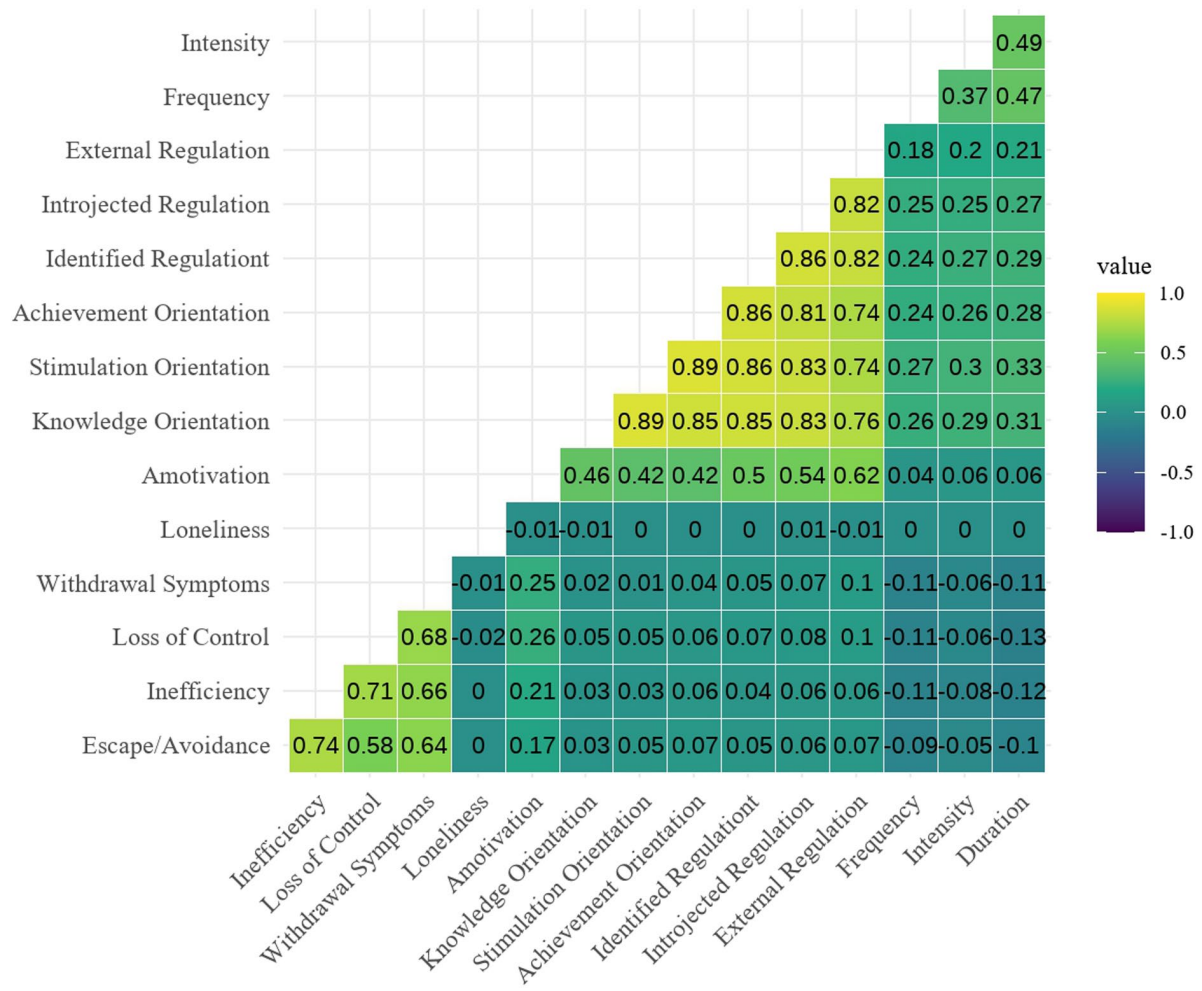
Correlation coefficient diagram.

Figure 3 illustrates the correlation heatmap among the research variables. Within the physical activity dimensions, intensity, duration, and frequency demonstrate moderate positive associations, reflecting the structural consistency inherent in exercise behavior. The dimensions of smartphone addiction are significantly and positively correlated with one another, while generally exhibiting negative associations with physical activity indicators; this suggests that higher levels of smartphone dependency correspond to lower engagement in physical exercise. Regarding sport motivation, all dimensions—with the exception of amotivation—show significant positive correlations, with particularly close links observed between knowledge, achievement, and stimulation orientations. Conversely, amotivation is significantly and negatively associated with both physical activity and positive motivational dimensions. Notably, loneliness exhibits a positive association with smartphone addiction dimensions (such as escape) while showing negative correlations with both physical activity and positive sport motivation. This result reveals a potential link for loneliness between the addiction system and the health behavior system. Overall, physical activity is positively associated with positive sport motivation, whereas smartphone addiction shows a clear negative association with both. This complex interactive relationship establishes an empirical foundation for identifying core nodes and intervention targets through subsequent network analysis.

### 4.2 Network structure analysis

#### 4.2.1 Network structure results analysis of each index

**Figure 4:**
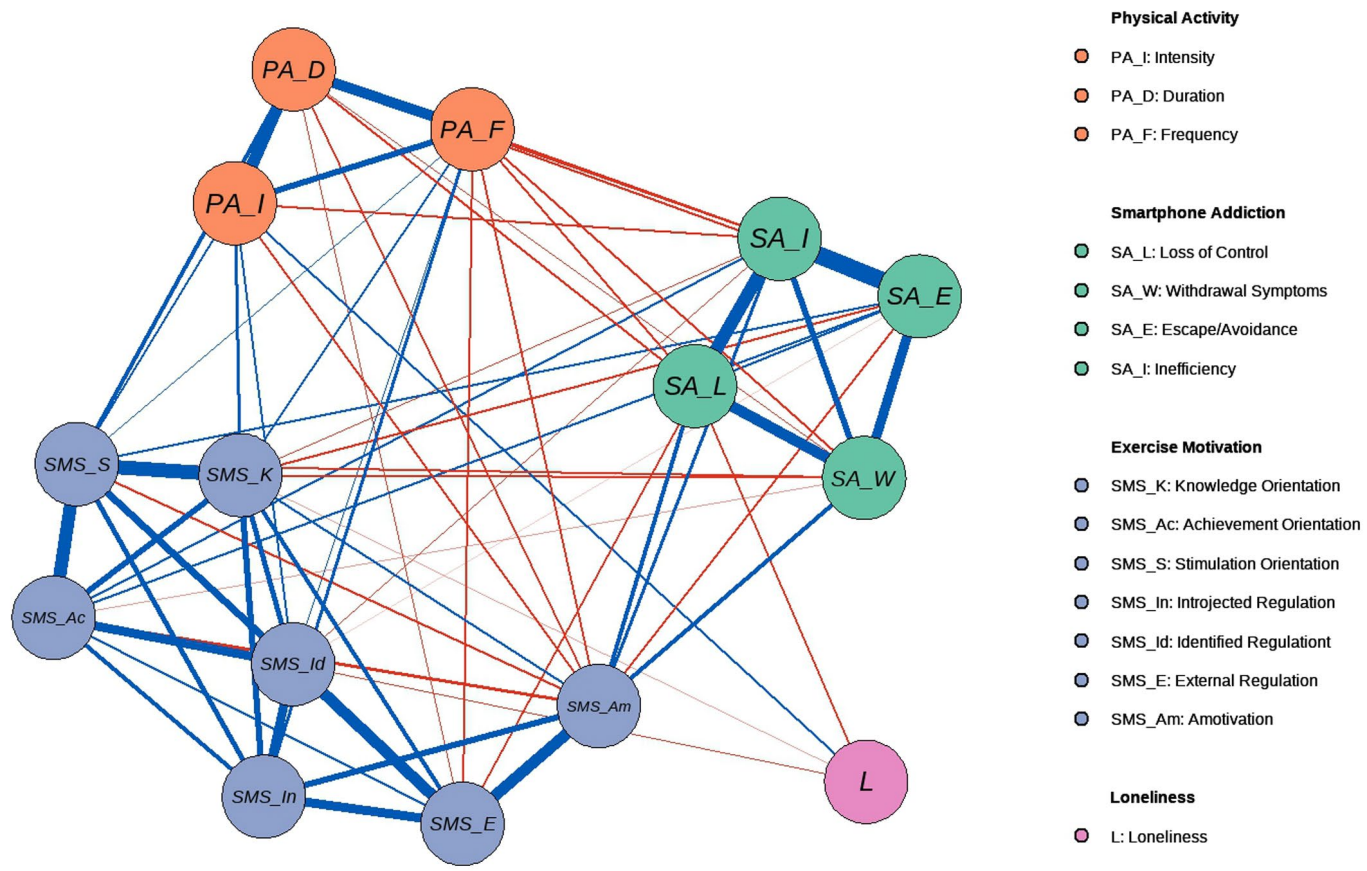
Network structure diagram.

Figure 4 illustrates the topological structure of the psychological network among the study variables. The results reveal complex and non-linear interactions between physical activity, smartphone addiction, sport motivation, and loneliness. The network structure displays three internally robust clusters: the physical activity cluster (duration, intensity, and frequency), the smartphone addiction cluster (loss of control, withdrawal, escape, and inefficiency), and the sport motivation cluster (knowledge, achievement, stimulation, introjection, identification, external regulation, and amotivation). Positive edges between nodes reflect the high degree of internal consistency within each cluster. Regarding cross-domain associations, sport motivation is positively associated with physical activity, revealing a close link between motivational resources and engagement in healthy behaviors. Within the context of Chinese collectivist culture, this association often involves the alignment of social norms with personal achievement. Conversely, extensive negative edges exist between smartphone addiction and physical activity, corresponding to the association between high smartphone dependency and low exercise participation. Notably, the “Amotivation” node shows relatively weak associations within the motivation cluster but exhibits significant cross-system pathways with various smartphone addiction nodes. This finding reveals the pivotal role of amotivation as a bridge node, which co-occurs with the specific psychological vulnerabilities observed in China’s “only-child” generation when facing digital temptations. Furthermore, loneliness, as an independent node, is associated with exercise frequency and autonomous motivation through negative edges. In the context of digital campus culture, this association reflects the antagonistic relationship between negative affect and health-promoting factors. In summary, the network structure not only identifies core nodes maintaining system stability but also reveals deep association patterns between smartphone addiction, motivational depletion, and physical inactivity through cross-cluster edges. These findings provide an empirical basis for the development of multi-dimensional systemic intervention strategies.

#### 4.2.2 Network structure centrality index analysis

**Figure 5:**
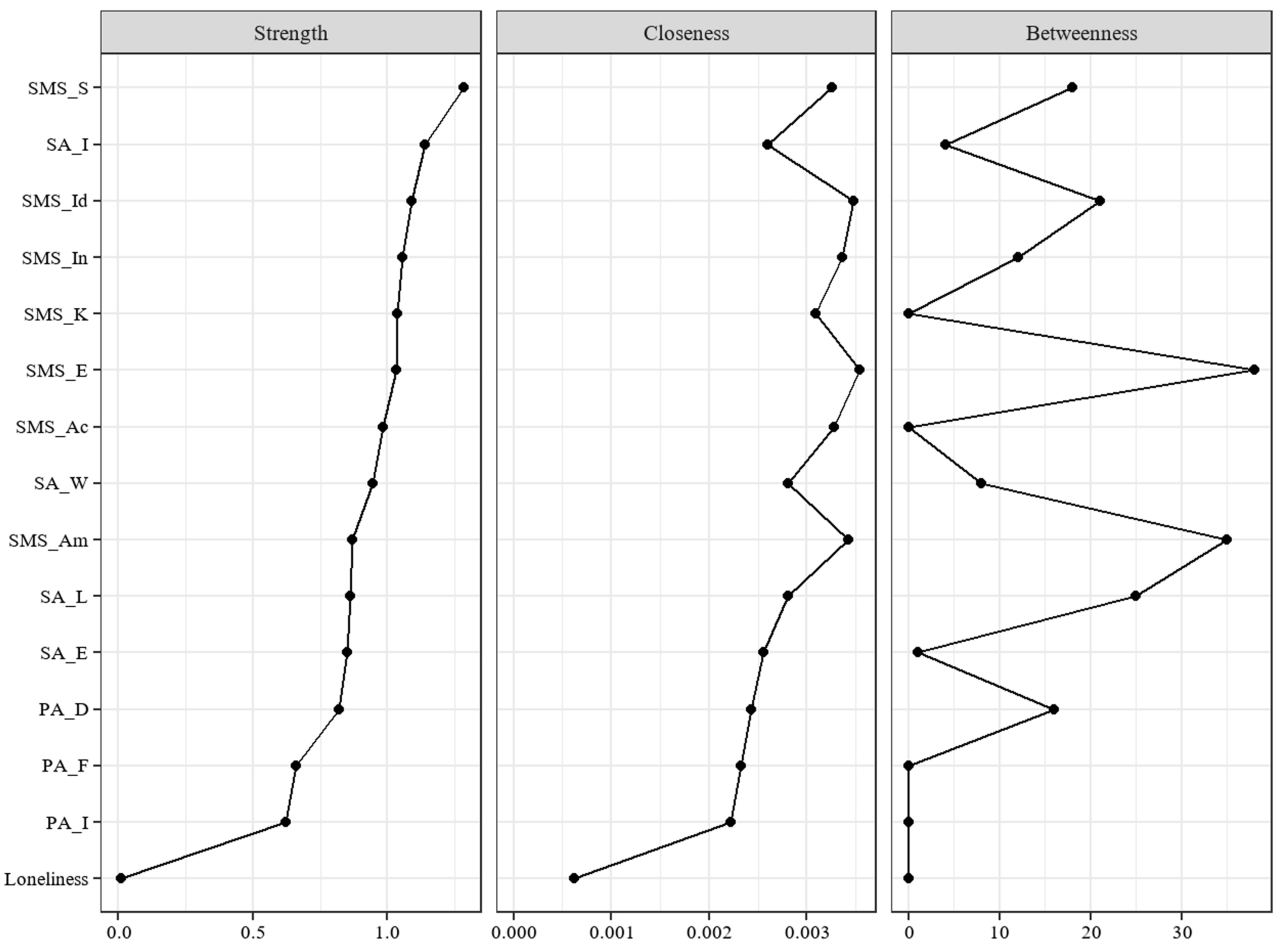
Schematic diagram of network structure centrality and three panels.

Figure 5 illustrates the three core metrics of network centrality—Strength, Closeness, and Betweenness—reflecting the functional roles of various variables within the complex interactive system. Within the sport motivation domain, stimulation orientation, identified regulation, and introjected regulation yielded high scores in Strength and Closeness. This reveals a high degree of association between these motivational components and the overall network connectivity and stability.

In terms of Betweenness centrality, significant peaks were observed for external regulation, amotivation, and the “loss of control” dimension of smartphone addiction. These results correspond to the critical “gatekeeping” roles these variables play in cross-system information transmission. Specifically, amotivation and loss of control function as pivotal hub nodes, linking the positive sport motivation cluster with the negative smartphone addiction cluster. Conversely, all centrality metrics for loneliness and exercise intensity were relatively low, indicating their peripheral positions within the current network structure. In the context of digital campus culture, these distributional characteristics highlight the close link between the sport motivation system and network stability. Furthermore, specific non-autonomous motivational components, together with smartphone addiction traits, constitute key pathways for cross-domain interaction. These findings provide an empirical reference for identifying structural targets for behavioral interventions.

Table 4 presents the top five positive and negative edge weights in the regularized partial correlation network, revealing the association patterns among variables. Strong positive associations primarily exist within the same domains, such as the link between escape-oriented use and inefficiency in smartphone addiction (0.50), as well as among stimulation, knowledge, and achievement orientations in sport motivation (0.40–0.41). These results reflect a high degree of structural consistency among the indicators. Regarding cross-system associations, physical activity indicators generally exhibit negative associations with smartphone addiction and amotivation. For instance, negative links exist between exercise duration and loss of control symptoms (−0.04), exercise frequency and withdrawal symptoms (−0.03), and exercise intensity and amotivation (−0.03). These results correspond to the synchronous associations among the related variables. Within the context of digital campus development in Chinese universities, lower levels of physical activity are associated with higher traits of smartphone dependency, while the weakening of sport motivation corresponds to increased levels of loss of control in smartphone use and loneliness.

**Table 4:** Edge weights between variables.

Var1	Var2	Weight
SA_EA	SA_Ineff	0.49
SMS_Know	SMS_Stim	0.41
SA_LoC	SA_Ineff	0.40
SMS_Ach	SMS_Stim	0.39
PA_Int	PA_Dur	0.37
SMS_Ach	SMS_Amot	−0.05
PA_Dur	SA_LoC	−0.04
SMS_Stim	SMS_Amot	−0.04
PA_Int	SMS_Amot	−0.03
PA_Freq	SA_WS	−0.03

These association pathways reveal the potential interconnected mechanisms among addictive behavior, motivational deficit, and healthy behavior. Particularly within the “only-child” generational background, addiction traits and negative affect may relate to lower exercise enthusiasm within the same complex interactive network. Such associations provide an important reference for future longitudinal studies to further verify the directional relationships between variables.

#### 4.2.3 Network stability analysis

**Figure 6:**
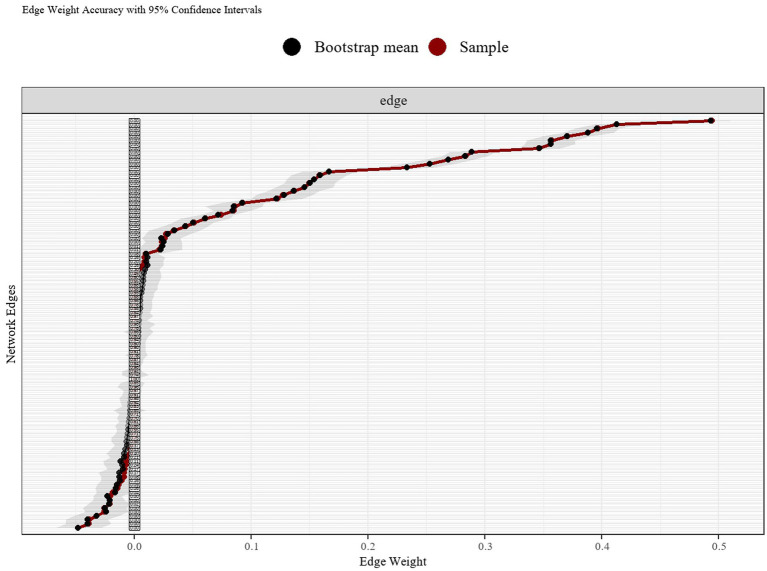
Visualizing edge confidence intervals for network structures.

The results of the edge confidence intervals obtained through the bootstrapping procedure are presented in Figure 6. The sample estimates are largely consistent with the bootstrap means, showing only minimal differences. Most edges exhibit narrow confidence intervals, indicating high accuracy and stability of the estimates. Moreover, the confidence intervals for stronger edges are relatively smaller, whereas those for weaker edges are slightly wider—this pattern aligns with common characteristics observed in network analysis, suggesting that the overall estimation of the network structure is reliable.

Table 5 illustrates the analysis results of the centrality stability coefficients for the psychological network (*N* = 10,676). The CS-coefficients for Strength and Closeness both reached the upper test limit of 0.75, indicating an extremely high level of stability. The CS-coefficient for Betweenness was 0.439, significantly exceeding the 0.25 threshold recommended by Epskamp et al., thereby meeting the reliability standards for academic research. Furthermore, the full-sample bootstrapping test (with 1,000 iterations) confirmed the statistical robustness of the indicator rankings within the network’s topological structure. These findings provide a solid empirical foundation for the subsequent identification of core hubs and bridge nodes within the network.

**Table 5:** Centrality stability coefficients (CS-coefficients) for the Psychological Network (N = 10,676).

Centrality index	CS-coefficient (*r* = 0.7)	Threshold (>0.25)	Interpretation
Strength	0.75	Pass	Highly stable
Closeness	0.75	Pass	Highly stable
Betweenness	0.439	Pass	Stable/reliable

**Figure 7:**
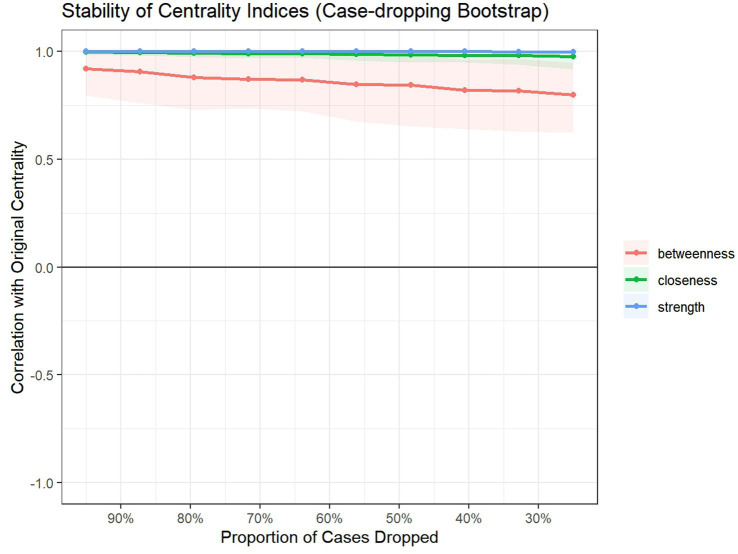
Calculate the stability coefficient of central index.

The stability testing results based on the case-dropping bootstrap are illustrated in Figure 7, demonstrating that the centrality indices within the psychological network exhibit ideal robustness. The stability curves for Strength and Closeness remained near 1.0 even as the proportion of dropped cases increased significantly, reflecting an exceptionally high level of structural stability. Although Betweenness showed slight fluctuations as the drop proportion increased, its correlation coefficient remained consistently high. Furthermore, the resulting Centrality Stability coefficient (CS-coefficient) was 0.439, significantly exceeding the academic threshold of 0.25. This comprehensive analysis confirms the consistency of the network’s topological attributes across different sample scales, thereby ensuring the empirical validity for the subsequent identification and interpretation of core hub nodes.

#### 4.2.4 Community detection analysis of network structure

In this study, the Louvain algorithm was employed to perform an in-depth analysis of the modular features of the network structure. The results show that the modularity index (Q) of the partition scheme is 0.458, which is significantly higher than the standard threshold of 0.3. This indicates that the network possesses a clear and robust community structure.

Furthermore, to verify the optimality of the partition scheme, the Walktrap algorithm was introduced for cross-validation. Both independent algorithms identified three consistent communities: the physical activity community, the smartphone addiction community, and the sport motivation and loneliness community. These findings provide strong evidence for the statistical stability and scientific validity of the observed topological structure.

**Figure 8:**
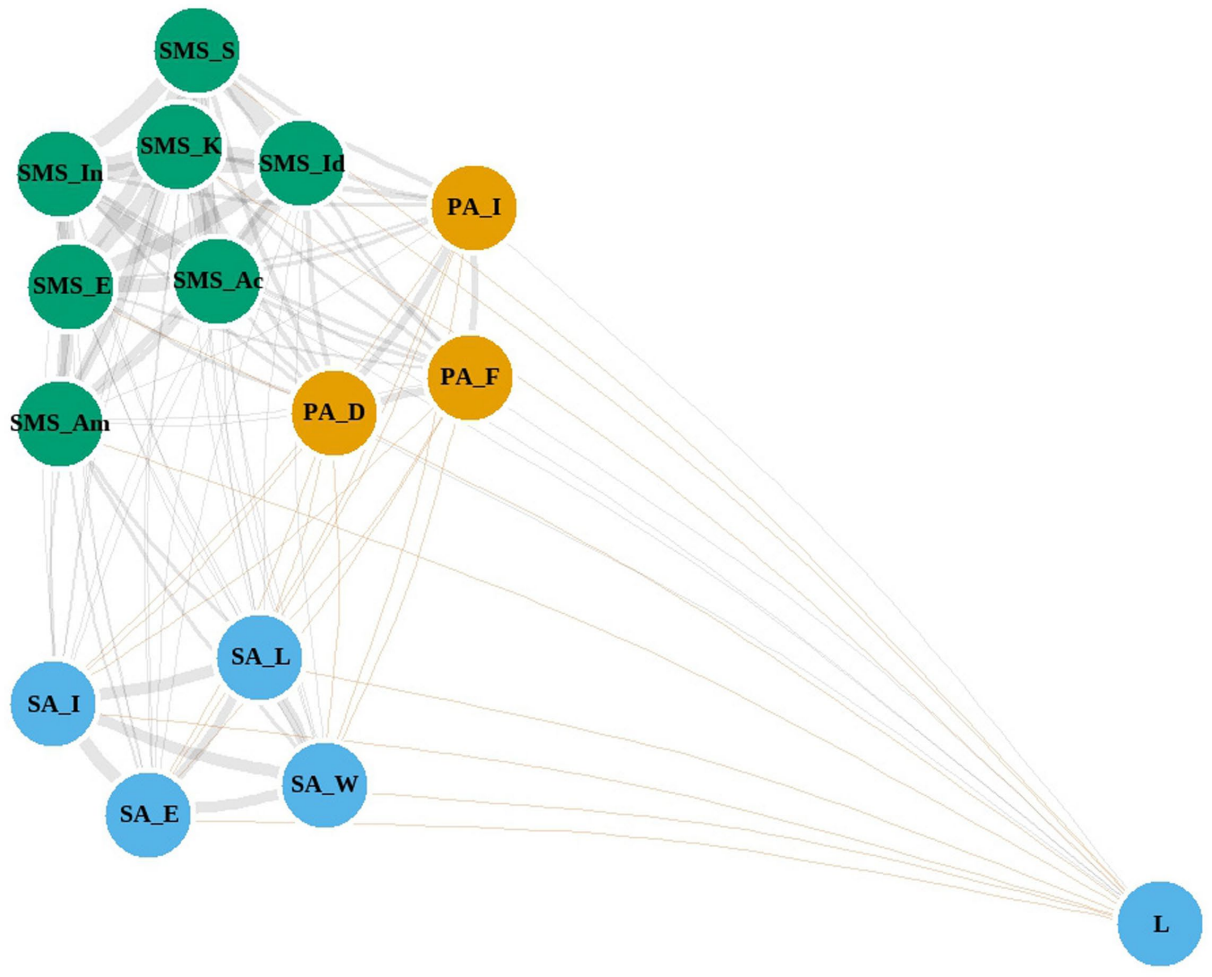
Network structure community detection.

Figure 8 illustrates the modular distribution of variables based on the Louvain algorithm. Verified by 1,000 bootstrap resamplings (95% case-drop bootstrap), the network exhibited a highly robust three-community structure with a stability of 99.8%. The system was partitioned into three functional clusters with high internal consistency. The “Physical Activity Community,” comprising exercise intensity, duration, and frequency, demonstrated clear boundaries, reflecting the structural independence of health behaviors within the system. The “Psychological Negativity Community” consisted of smartphone addiction and loneliness; this cluster encompassed core addiction dimensions such as loss of control and escape behaviors, with loneliness showing high integration into this cluster during statistical evolution. This suggests that loneliness is not only a trigger for smartphone addiction but also forms a tight psychopathological complex with addictive symptoms within the topological structure. The third cluster, the “Sport Motivation Community,” included variables such as knowledge, achievement, and stimulation orientations, constituting the dynamic core of the entire system. Notably, although loneliness occasionally showed an independent trend in full-sample analysis, its marginal distribution characteristics and close link with Amotivation revealed its “bridge” role in connecting diverse functional mechanisms. Furthermore, the algorithmic consistency test showed that the results of the Louvain and Walktrap algorithms were perfectly aligned (ARI = 1.00), further confirming the objectivity and robustness of the three-community partition. Overall, the community detection results not only confirm the structural independence of each behavioral construct but also provide critical empirical evidence for understanding the stable interaction logic between variable clusters through the marginal positioning and functional integration of loneliness.

#### 4.2.5 Bridging centrality analysis

**Figure 9:**
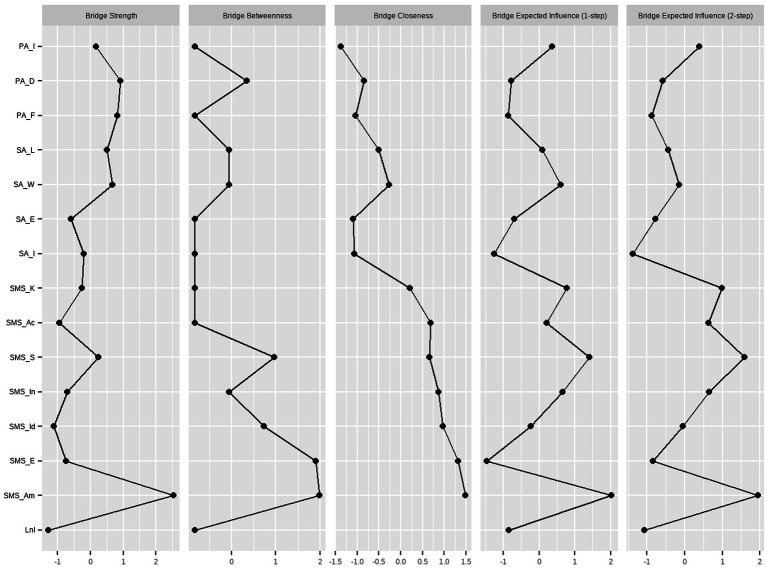
Bridge centrality metrics of network nodes across variable communities.

Figure 9 illustrates the results of the bridge centrality analysis across variable clusters, identifying key hubs that connect different psychological and behavioral domains. Among all nodes, amotivation exhibits the most significant bridge attributes. Amotivation ranks first in bridge strength, bridge betweenness, and bridge expected influence (2-step), corresponding to its mediating role between the sport motivation cluster and the smartphone addiction community. This high degree of bridge association reflects a specific psychological coupling between motivational deficit and addictive behavior when individuals face digital temptations.

Furthermore, stimulation orientation and knowledge orientation within the sport motivation cluster yield high scores in multi-level bridge influence, revealing a close link between intrinsic motivational components and both exercise behaviors and psychological traits. Within the smartphone addiction dimensions, loneliness and withdrawal symptoms demonstrate high cross-cluster associations, involving potential functional pathways between addiction symptoms and the physical activity community. These associations reflect the interconnectedness between smartphone usage norms and socio-emotional needs within the digital campus culture of Chinese universities.

**Figure 10:**
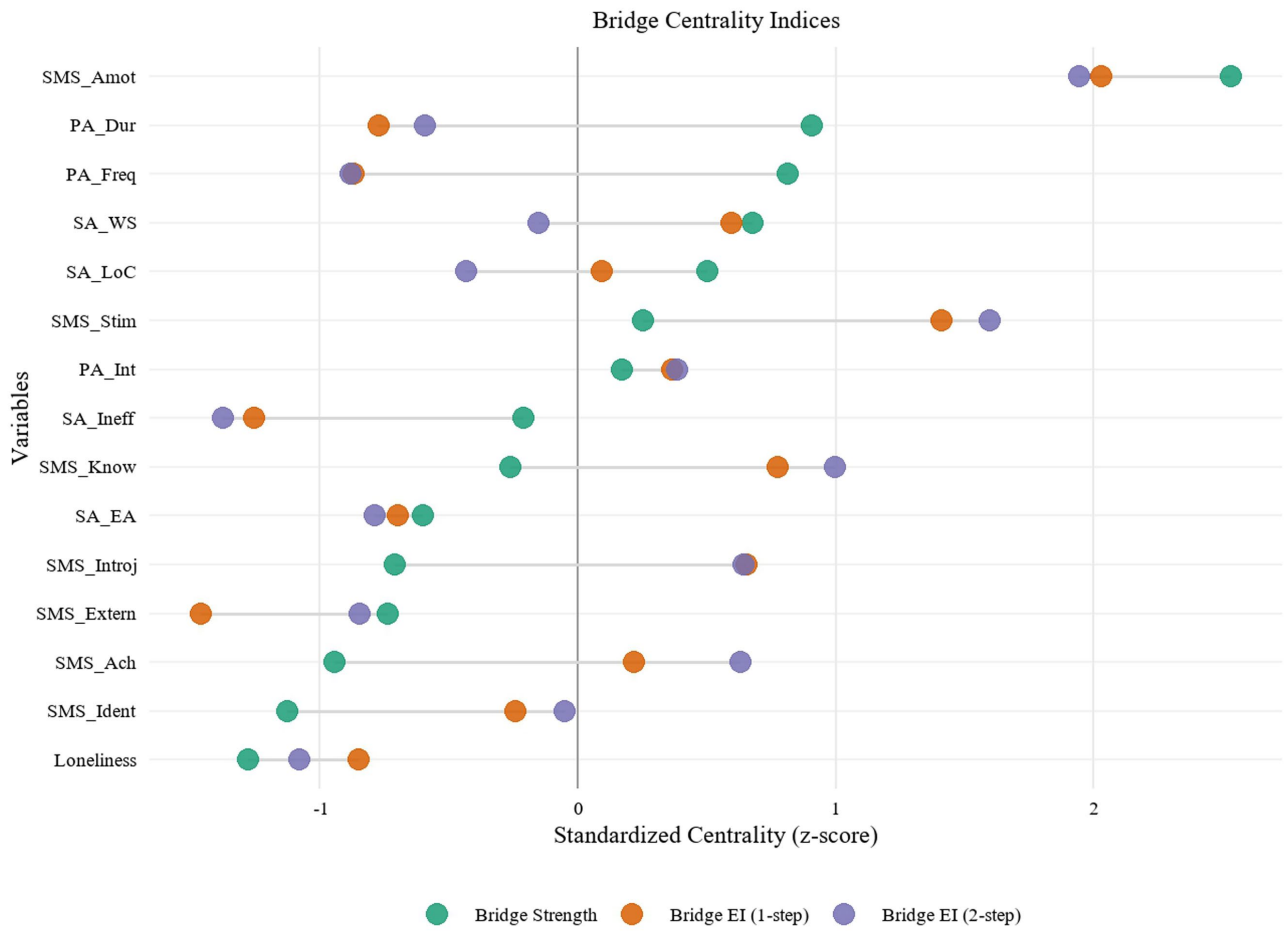
Diagram of bridge centrality.

Figure 10 illustrates the analysis results of bridge centrality metrics, identifying the key hub nodes that connect distinct psychological and behavioral domains within the network system. Among all nodes, amotivation exhibits the most prominent bridge attributes, ranking first in both bridge strength and bridge expected influence (2-step). This underscores its core mediating role in linking the sport motivation cluster with the smartphone addiction community. Within the sport motivation domain, stimulation orientation and knowledge orientation yield high scores in both first-order and second-order bridge influence. This indicates that intrinsic motivational components play a vital role in associating exercise behavior with psychological traits. Regarding the smartphone addiction dimensions, loneliness and withdrawal symptoms demonstrate high cross-cluster association characteristics, serving as potential functional pathways between addiction symptoms and the physical activity community.

In contrast, variables such as identified regulation and escape orientation show lower bridge centrality, reflecting their peripheral status in inter-community interactions. Overall, the bridge centrality analysis reveals the cross-domain coupling characteristics of amotivation and specific intrinsic motivation dimensions within the network. These findings provide critical structural evidence for understanding the complex interactions between addiction, affect, and health behaviors within a non-causal topological framework.

#### 4.2.6 Comparison of gender-disaggregated networks

**Figure 11:**
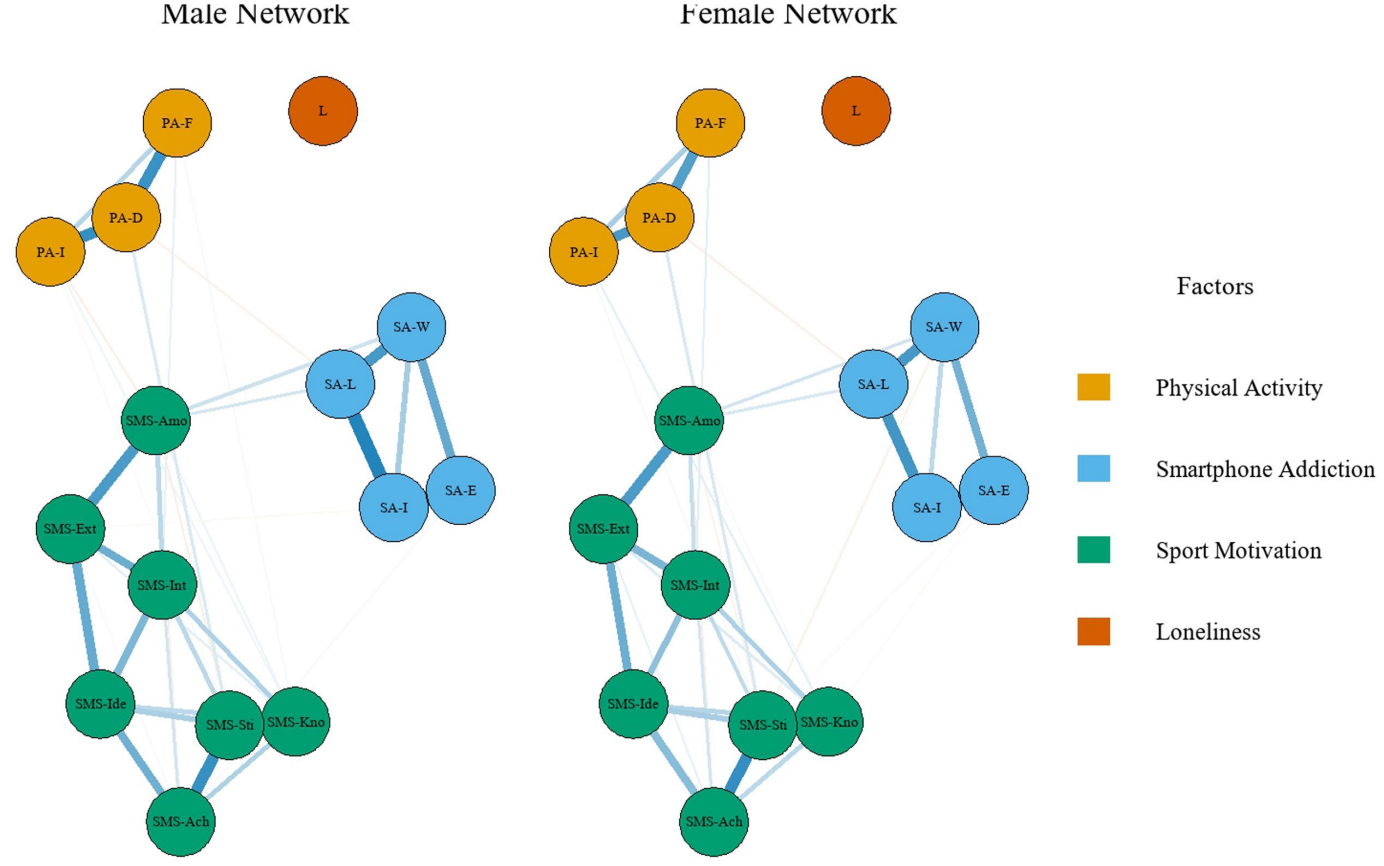
Comparative psychological networks of physical activity, smartphone addiction, sport motivation, and loneliness by gender.

Figure 11 illustrates the network topological structures of physical activity, smartphone addiction, sport motivation, and loneliness for both male and female groups. Although both groups exhibit similarities in clustering characteristics, the Network Invariance Test reveals significant gender differences. At the local edge level, these differences correspond to gender-specific interaction patterns.

Within the sport motivation domain, the association between “knowledge orientation” and “achievement orientation” is significantly stronger in the female group than in the male group. In the Chinese cultural context, female self-achievement relates highly to the internalization process of exercise knowledge; conversely, this association is more loosely connected in males, whose motivation involves diverse social expectations such as competition and socialization. Furthermore, the node centrality for physical activity (e.g., PA_Dur) is significantly higher in males than in females. This reflects a stronger association between male exercise habits and core elements such as rules and routines, whereas female exercise participation relates more to environmental support or spontaneous social factors, corresponding to lower centripetal force within the network.

Regarding cross-dimensional associations, the link between physical activity and smartphone inefficiency is closer in the male group, corresponding to a higher relevance of physical activity as a potential replacement mechanism for smartphone use among males. In the addiction domain, the association between “escape” and “inefficiency” is stronger in females, which relates to the psychological trait of using digital channels for emotional regulation when facing stress. These findings provide important evidence for understanding gender-specific intervention mechanisms within the Chinese cultural context.

The results of the Network Comparison Test (NCT) are presented in Table 6, revealing statistically significant differences between gender groups across six specific edges. The most pronounced difference was observed in the link between Loss of Control (SA_L) and Achievement Motivation (SMS_Ac) (*p* = 0.0004). Significant paths were also identified between Withdrawal Symptoms (SA_W) and Stimulation Orientation (SMS_S), as well as between Knowledge Orientation (SMS_K) and Stimulation Orientation (SMS_S). Furthermore, the edge weights connecting Physical Activity Intensity (PA_I) with two regulation dimensions (SMS_In, SMS_E) exhibited gender heterogeneity. The identified significant edge differences (Δ*w*) ranged from 0.0029 to 0.0453. Although the effect sizes were relatively small, the massive sample size amplified the statistical power to detect subtle variations. Therefore, rigorous permutation tests were employed to ensure the robustness of these differences. These six divergent paths reflect nuanced adjustments in interaction patterns across genders rather than structural fractures. The results suggest minor structural propensity differences between sport motivation and smartphone addiction across genders. However, to further clarify the practical transformational capacity of these micro-weight differences in intervention effects, longitudinal tracking experiments are warranted for future validation.

**Table 6:** Summary of significant edge differences and effect sizes between genders via Network Comparison Test.

Edge comparison (Node A–Node B)	Effect size (∣Δ*w*∣)	*p*-value	Significance
SA_L (Loss of Control)–SMS_Ac (Achievement)	0.015	0.0004	***
SA_W (Withdrawal)–SMS_S (Stimulation)	0.0317	0.0072	**
SMS_K (Knowledge)–SMS_S (Stimulation)	0.0453	0.0324	*
PA_I (Intensity)–SMS_In (Introjected)	0.0102	0.0492	*
SA_W (Withdrawal)–SMS_Id (Identified)	0.0029	0.0452	*
PA_I (Intensity)–SMS_E (External)	0.0117	0.0264	*

|Δ*w*| represents the absolute difference in edge weights between the male and female networks. Significance levels: ^*^p < 0.05, ^**^p < 0.01, ^***^p < 0.001.

**Figure 12:**
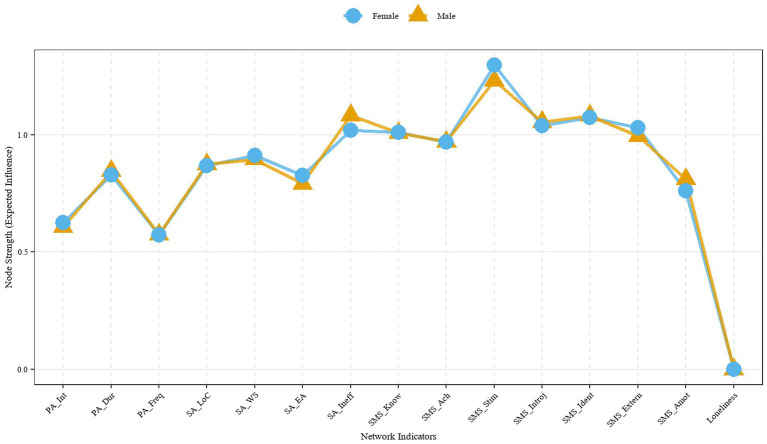
Centrality comparison (strength) between male and female networks.

Figure 12 illustrates the distribution of Expected Influence (EI) across genders. Overall, the networks for both groups exhibit a high degree of synchrony, reflecting the stability of psychological structures across genders. Within the sport motivation domain, “Stimulation Orientation” (EM_Stim) remains central in both groups, whereas “Loneliness” corresponds to the periphery of the network.

Data analysis reveals that the strength of physical activity dimensions (e.g., PA_Dur and PA_Freq) is significantly higher in males than in females. In the Chinese social context, this characteristic corresponds to the stronger “structure” and “persistence” of male exercise habits, with their exercise behaviors demonstrating higher associative efficacy within the network. In contrast, female exercise participation relates to more environmental or spontaneous factors, corresponding to a slightly weaker centripetal force in the network. Furthermore, the high intensity of “Amotivation” (EM_Amot) in males relates to negative psychological associations within their behavioral patterns. Regarding motivational regulation, females show a slight lead in centrality for “External Regulation” and “Identified Regulation,” reflecting a higher correlation between female motivational transformation and social evaluation or value identification.

#### 4.2.7 Global and structural network comparison

**Figure 13:**
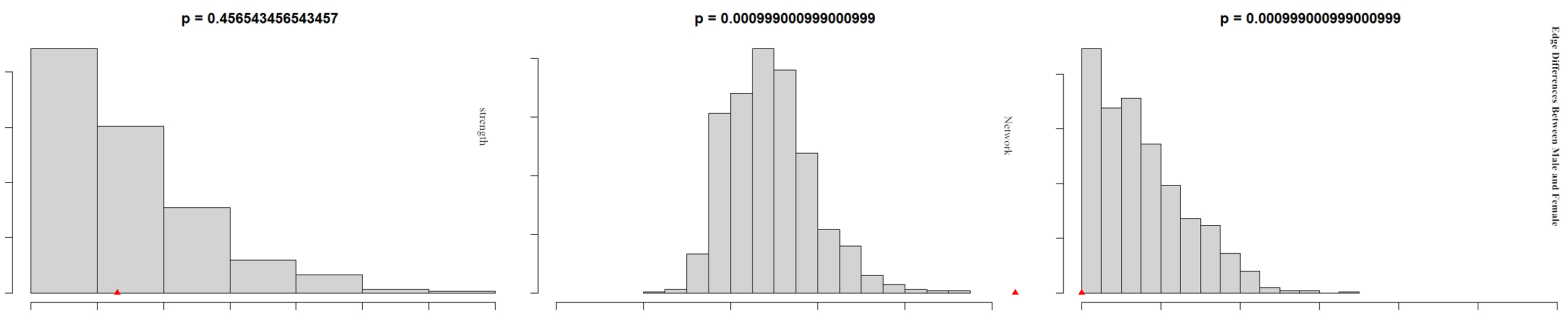
Results of gender group network comparison.

Figure 13 presents the results of the Network Comparison Test (NCT). The findings indicate no significant difference in Global Strength between the male and female groups (*p* ≈ 0.456), suggesting that the overall connectivity density of the networks is consistent across genders.

However, the Network Structure Invariance test reveals significant topological differences (*p* < 0.001), reflecting gender-specific patterns in the relationships between variables. Further tests on edge weight differences show that the key connections between sport motivation and smartphone addiction deviate significantly between genders (*p* < 0.001). In summary, while the global strength of the networks is comparable between genders, the internal structural organization involves different psychological mechanisms. These results provide empirical evidence for understanding gender-specific behavioral habits.

### 4.3 Summary of hypotheses testing

Table 7 summarizes the results of the verification of the three preset hypotheses using Psychological Network Analysis (PNA). The findings indicate that all three hypotheses are supported by the empirical data.

**Table 7:** Summary of hypotheses testing results.

Hypothesis	Specific path/Analysis	Key statistical indicators	Result
*H1*: Intrinsic motivation facilitates PA.	Edge: EM_Stim, EM_Ach → PA_Int, PA_Dur	Edge weights ≈ 0.15–0.33; High Strength Centrality	Supported
*H2*: Loneliness suppresses PA via SA.	Bridge Edges: Loneliness → SA_EA; SA → PA	Negative partial correlations; High Bridge Centrality of Loneliness	Supported
*H3*: Amotivation acts as a cross-system bridge.	Bridge Centrality (Strength, Betweenness)	EM_Amot ranked 1st in Bridge Strength and 2-step EI	Supported

Specifically, stimulation orientation and achievement orientation within the sport motivation cluster exhibit significant positive edge weights (0.15–0.33) with physical activity (intensity and duration), while also demonstrating high strength centrality. This result confirms the core facilitative role of autonomous motivation within the network (H1). In terms of cross-system associations, loneliness demonstrates its role as a bridge risk factor between the addiction and health behavior systems through its positive association with the escape dimension of smartphone addiction and the general negative partial correlations between smartphone addiction and physical exercise participation (H2). Furthermore, amotivation (EM_Amot) ranks first in the network across bridge strength, bridge betweenness, and bridge expected influence (2-step). This precisely identifies its topological status as the core mediator connecting the sport motivation cluster and the smartphone addiction community (H3).

## 5 Discussion

### 5.1 The strategic architecture of sport motivation: stability hubs and bridge vulnerabilities

The topological architecture of the network in this study reveals the dual attributes of the sport motivation system in being associated with the stability of health behaviors and serving as a bridge for potential risk co-occurrence. Firstly, the significant positive edge weights between autonomous motivation nodes (e.g., EM_Stim, EM_Ach) and physical activity (PA), along with their leading strength centrality metrics, provide strong empirical support for the core hypotheses of Self-Determination Theory (SDT; H1). These intrinsic motivational dimensions serve not only as the homeostatic core of the health behavior system but also as the structural power source for maintaining exercise persistence among university students (Deci and Ryan, 2020; Morouço et al., 2025). Notably, unlike the Western individualistic context that emphasizes personal interest, sport achievement among Chinese university students under a collectivist culture is often deeply coupled with fulfilling social responsibilities and meeting group expectations. This goal-oriented cultural trait enhances the cohesion of motivational nodes within the network, making them key hubs for connecting health behaviors.

However, bridge centrality analysis further identifies “Amotivation (EM_Amot)” as the most prominent cross-domain vulnerability within the system, ranking first in both bridge strength and two-step expected influence. This finding transforms the motivational regulation theory of SDT from a macro-description into a micro-topological explanation: the inhibition of exercise behavior by smartphone addiction is not a simple direct exclusion but is realized through a risk spillover in a state of “motivational vacuum” (H3). When individuals are in a state of amotivation, the self-regulatory system collapses, acting as a “gateway” for the addiction system to permeate the health behavior system. This complements the perspective of Van Borkulo et al. (2023), who proposed that amotivation serves as an “affective attractor” in internet addiction networks. Particularly among the “only-child” generation in China, the lack of direct peer interaction support makes individuals highly susceptible to self-regulatory dysfunction when facing digital temptations, leading to the strong cross-system coupling of the amotivation node.

By comparing different centrality metrics, it is evident that the “stability hubs” (e.g., EM_Stim) responsible for maintaining behavioral consistency and the “bridge hubs” (e.g., EM_Amot) responsible for risk diffusion are distinct variables. This functional separation provides a critical structural reference for precision interventions: preventive strategies should aim to strengthen high-strength autonomous motivational “cores” to enhance overall systemic resilience; conversely, restorative interventions should focus on blocking the “amotivation” bridge node to intercept the pathological spread of smartphone addiction symptoms into the sports domain. By precisely targeting bridge variables, researchers can not only restore the exercise dynamic system but also effectively curb the cross-system spread of addictive emotions within the psychological network.

### 5.2 The affect-addiction Axis: loneliness-driven compensatory pathways to physical inactivity

Network topology analysis identified a robust cross-community pathway—namely, “Loneliness–Smartphone Addiction (Escape)–Physical Activity”—providing topological evidence for understanding the potential association patterns of health behaviors among university students (H2). Correlation heatmaps and edge weights showed a significant positive connection between loneliness and the “escape behavior (SA_EA)” dimension of smartphone addiction, while a structural negative association was observed between smartphone addiction and physical activity (PA). This result aligns closely with the theoretical framework of the I-PACE model, which posits that individuals facing negative emotional stress (such as loneliness) tend to engage in compensatory behaviors for emotional regulation, which is associated with the co-occurrence of addiction cycles and executive function deficits (Brand et al., 2016; Mehmood et al., 2021).

Within the specific context of “digital campuses” in Chinese universities, this structural negative correlation reflects an antagonistic relationship between excessive digital dependency and health behaviors. As smartphones have become the core medium of campus life, high-dependency environments often lead to a blurring of the boundary between normal use and over-reliance. Campus social pressure prompts some students to utilize smartphones as a “safe haven” against real-life loneliness; the high-weight connection between “escape-oriented use (SA_EA)” and loneliness in the network is a manifestation of this “emotional hedging” behavior. Consistently, recent findings indicate that physical activity may serve as a critical mediator between problematic internet use and negative emotions among university students, providing an additional empirical anchor for the I-PACE components in our model (Jelleli et al., 2024). This compensatory logic, based on a specific campus culture, may explain the negative link between loneliness and real-world exercise through digital channels.

Furthermore, as a bridge variable, loneliness precisely points toward the symptom node of “escape-oriented use” rather than being uniformly distributed across all addiction dimensions. This finding refines the traditional view of loneliness as a “generalized driver,” suggesting instead that it primarily relates to specific addictive symptoms through “emotional hedging.” Compared to some Western studies (Karska et al., 2023), this research highlights the factor of social withdrawal within the Chinese cultural context: loneliness often co-occurs with a decrease in collective exercise participation, making it a systemic interference factor for healthy behaviors. Therefore, we hypothesize that precision interventions might consider targeting the micro-pathway of “Loneliness–Escape Motivation,” potentially blocking the association of risks from the emotional system to the behavioral system by alleviating real-life social isolation (Xu et al., 2024; Liu et al., 2025).

### 5.3 System robustness and gendered topologies: implications for targeted interventions

Bootstrap stability analysis verified the reliability of edge weights and centrality rankings, providing solid methodological support for the research findings. On the basis of ensuring a robust network structure, the Network Comparison Test (NCT) revealed significant topological heterogeneity between gender subgroups. Although no significant difference was observed in global strength between male and female students, the weight distribution of micro-pathways exhibited distinct gender-specific characteristics. In the female subnetwork, stronger edge weights were observed between smartphone addiction (particularly the escape dimension, SA_EA), sport motivation, and loneliness, reflecting a greater tendency among females to utilize digital media as a “channel for emotional regulation” under pressure. This finding echoes the conclusion of Zhou and Feng (2025), suggesting that the emotional dependency of female students is a key systemic factor leading to the decline of their sport motivation.

In contrast, the male subnetwork demonstrated a stronger logic of behavioral consistency. Males exhibited more concentrated connections at physical activity nodes, and their exercise behaviors were relatively less interfered with by addiction variables. This difference reflects that, under the influence of cultural backgrounds and gender roles, males tend to maintain health behavior systems through behavioral logic (such as habit consolidation and social reinforcement), whereas females are more susceptible to motivational impairment driven by “emotional hedging.” Although the core hypotheses of this study were supported in the full sample, these gendered local effects—such as the stronger association with emotional escape in the H2 pathway for females and the higher behavioral connectivity in the H1 pathway for males—refine the universality of the global hypotheses and reveal subtle heterogeneous features within the system.

Based on these findings, this study proposes gender-sensitive precision intervention strategies. For female students, the focus of intervention should be placed on emotional regulation and psychological support to reduce their demand for “emotional hedging” via digital channels by alleviating real-life social pressure, thereby restoring sport motivation. For male students, the intervention emphasis should shift toward the consolidation of behavioral habits and social reinforcement; leveraging their stronger behavioral connectivity, collective sports competitions can be utilized to enhance the centripetal force of their sport motivation. This transformation from “topological differences” to “categorized policies” not only improves the specificity of interventions but also provides a theoretical basis for universities to develop differentiated behavioral intervention frameworks in the digital age.

### 5.4 Theoretical contributions

This study deepens the understanding of the interaction mechanisms between psychology and behavior among university students through Psychological Network Analysis (PNA). Its theoretical contributions are primarily reflected in the following three aspects. It achieves the integration of the empirical paradigms of Self-Determination Theory (SDT) and the I-PACE model. The study identifies “cross-system interfaces” within the network topology, demonstrating that the addiction evolution process in the I-PACE model (associated with loneliness) essentially involves the depletion of an individual’s basic psychological needs and corresponds to motivational impairment under the SDT framework. This integration provides a unified logical explanation for the evolution of health behaviors in the digital age. It redefines the topological attributes of “Amotivation.” This research breaks through the traditional view that treats amotivation merely as a “lack of motivation.” Through bridge centrality analysis, it proves that amotivation (EM_Amot) is the most potent cross-system hub in the entire network, serving as a “gateway” for addiction risks to permeate the health system. This supplements the structural evidence of SDT within multi-variable interaction networks. It reveals the cultural specificity and structural stability of motivational constructs. The high strength centrality of autonomous motivation validates the cross-cultural universality of core SDT constructs. Meanwhile, the gender heterogeneity identified through the Network Comparison Test (NCT) provides empirical evidence for understanding the differential associations between “emotional hedging” and “behavioral persistence” among students of different genders within the Chinese cultural context.

### 5.5 Practical implications for intervention

Based on the identified core nodes and associative paths, this study proposes the following hypotheses to provide precise targets for improving college students’ mental health. Regarding cross-system decoupling, interventions should focus on controlling the “bridge hubs” of Amotivation and Loneliness rather than solely limiting screen time. University counselors should encourage social engagement to alleviate loneliness, thereby weakening the potential link between social isolation and “escape-oriented” social media use. In terms of motivation reshaping, given the central positions of Achievement Motivation and Stimulation Orientation in the network, physical education curricula should prioritize athletic challenges and enjoyment while de-emphasizing performance evaluation. Such pedagogical reforms could enhance the resilience of students’ psychological systems. Regarding gender differences, improving emotional regulation in female students may decouple the correlation of “emotional hedging” via digital channels. Conversely, for male students, consolidating behavioral habits and strengthening social identity through collective sports events could enhance the centripetal force of their exercise motivation.

### 5.6 Limitations and future directions

Despite the large-scale sample, this study is subject to several limitations.

(1) The cross-sectional design precludes the determination of causal directions. While the network topology reveals statistical associations, it cannot infer temporal evolutionary logic. Future research should employ longitudinal network models, such as Multilevel Vector Autoregressive (mlVAR) models, to further explore the dynamic coupling between smartphone addiction and sport motivation.
(2) Data collection relied primarily on self-report scales, which may be susceptible to social desirability bias. Future studies should integrate objective behavioral trajectories obtained from wearable devices (Pirwani and Szabo, 2025). Specifically, randomized controlled trials in athlete populations have confirmed that objective smartphone exposure significantly impairs cognitive performance, such as attention and reaction time (Dridi et al., 2026). Incorporating such objective metrics into experimental designs would provide a robust complement to self-reported psychological states and help verify the actual functional impairments caused by smartphone addiction in general student populations.
(3) The trade-off between statistical power and clinical significance must be considered given the substantial sample size (*N* > 10,000). In extremely large samples, minute edge differences (|Δ*w*||) easily reach statistical significance. Therefore, the gender-specific paths identified should be interpreted as structural tendencies with reference value rather than absolute intervention guides. Future research needs to further verify the transformational efficiency of these micro-weight differences in actual health behavior changes through effect-size benchmarking.
(4) There are limitations regarding cultural specificity and potential confounding variables. This study is rooted in the Chinese context. Given the differences between Eastern and Western cultures in sport motivation (e.g., sense of honor vs. hedonism) and social media use motives, cross-cultural research is urgently needed to verify the generalizability of this network topology in individualistic cultures. Furthermore, as network edges may involve unobserved confounders, subsequent longitudinal tracking should further verify the “bridge paths” to construct a more globally universal theory of health behavior associations.

## 6 Conclusion

Based on a Psychological Network Analysis (PNA) of 10,676 college students, this study confirms that physical activity, sport motivation, smartphone addiction, and loneliness constitute a highly robust and complex interactive system. The findings reveal that intrinsic motivation serves as the core driver for maintaining healthy behaviors, while “Amotivation” and “Loneliness” function as critical bridge hubs, mediating the cross-system percolation of addiction risks into the decline of exercise participation. Although the global strength of the networks remains consistent across genders, females exhibit more pronounced “emotional hedging” characteristics (loneliness-driven addiction), whereas males demonstrate higher behavioral consistency. Consequently, this study proposes that precision interventions should shift from traditional screen-time limitations toward the regulation of these “bridge nodes.” By alleviating social isolation, reshaping autonomous sport motivation, and implementing gender-specific strategies, such interventions can effectively block the spread of pathological symptoms and enhance the psychological and behavioral resilience of college students.

## Data Availability

The raw data supporting the conclusions of this article will be made available by the authors, without undue reservation.
