# Supplementary material for: The interrelationship among physical activity, smartphone addiction, loneliness, and sport motivation in Chinese college students: a cross-sectional network analysis
Source: Front Psychol. 2026 Jun 25;17:1720457. doi: 10.3389/fpsyg.2026.1720457 (PMC13346186; doi:10.3389/fpsyg.2026.1720457)
Supplement: Supplementary file 1 [file Supplementary_file_1.DOCX]

**Appendix**

**Research Tool** ：

The research design is based on the Self Determination Theory (SDT) and the Interaction of Person Affect Cognition Execution Model (I-PACE Model) (Elhai et al., 2022; Brailovskaia et al., 2023), combined with the Psychological Network Analysis (PNA) method to identify nonlinear relationship structures between variables (Epskamp et al., 2018).

To measure the core variables, four sets of standardized scales validated by Chinese soil chemistry were selected:

***Table 1 Overview and Psychometric Properties of the Selected Scales***

| Measuring variables using a scale | number of terms | Cronbach's α | cite |
| --- | --- | --- | --- |
| Physical Activity Rating Scale (PARS-3) | 3 | 0.797 | Zhang et al., 2022 |
| UCLA Loneliness Scale Version 3 | 20 | 0.846 | Lim et al., 2021 |
| Smartphone Addiction Scale (SAS) | 17 | 0.932 | Yang et al., 2023 |
| Sport Motivation Scale (SMS) | 28 | 0.967 | Zhang et al., 2023 |

The reliability of each scale is higher than the 0.70 standard, and the structural validity is good (all through exploratory factor analysis, KMO>0.73, Bartlett's sphericity test significant p<0.001). All four scales have been retested one month later and have good stability (r>0.87).

（1）Physical Exercise Scale

This study systematically evaluated participants 'exercise behaviors using the Physical Activity Rating Scale-3 (PARS-3) revised by Liang Deqing et al., which covers three dimensions: intensity, duration, and frequency (Zhang et al., 2022). To accurately capture different aspects of exercise behaviors, the study directly extracted three original items from the scale—rated on a 1-5 scale—as network nodes: physical activity intensity (PA_Int), duration (PA_Dur), and frequency (PA_Freq), rather than relying solely on total score weighting. Statistical analysis was performed using SPSS 26.0 and Amos 24.0. The results showed that the Cronbach's α coefficient of the scale in this study sample was 0.797. Confirmatory factor analysis (CFA) demonstrated good structural validity (χ²/df=3.29, RMSEA=0.032, GFI=0.921, CFI=0.913, IFI=0.938), with factor loadings ranging from 0.72 to 0.88. All original items were retained. The excellent reliability, validity, and structural stability of the PARS-3 scale provide scientific empirical support for further exploration of the relationships between physical exercise and mobile phone addiction, loneliness, and exercise motivation.

（2）UCLA Loneliness Scale (3rd Edition)）

This study employed the UCLA Loneliness Scale (3rd Edition) developed by Russell et al. (2021) to assess participants 'loneliness levels. The scale consists of 20 items scored on a 4-point scale, including 9 reverse-coded items that were re-coded according to recommendations, with higher scores indicating greater loneliness. During operationalization, the total scale score (Loneliness) was treated as a single node in network analysis. Statistical analyses demonstrated excellent reliability and validity of the scale in this sample, with a full-scale Cronbach's α coefficient of 0.905. Confirmatory factor analysis (CFA) yielded ideal results (χ²/df=2.18, RMSEA=0.021, GFI=0.951, FI=0.949, IFI=0.965), and all item standardized factor loadings were above 0.55. The scale's strong structural validity and high reliability provided a solid data foundation and empirical support for investigating the mediating relationship between physical exercise, mobile phone addiction, and loneliness.

（3）Smartphone Addiction Scale (SAS)

This study adopted the Smartphone Addiction Scale (SAS), a scale designed for Chinese college students, which consists of 17 items and uses a 5-point scoring system (Yang et al., 2023). During operationalization, the 17 items were aggregated into four network nodes through summation: SA_LoC (Loss of Control, 7 items), SA_WS (Withdrawal Symptoms, 4 items), SA_Ineff (Inefficiency, 3 items), and SA_EA (Avoidance, 3 items). A 5-point scoring system was used. If ≥5 items were selected as "often" or "always," the respondent was diagnosed with mobile phone dependence, with severity categorized into three levels (34-51 points, 51-68 points, 68-85 points). Statistical analysis demonstrated that the scale had good measurement reliability, with Cronbach's α coefficients for the four dimensions being 0.842,0.795,0.781, and 0.766, respectively. Confirmatory Factor Analysis (CFA) results confirmed excellent structural validity (χ²/df=2.31, RMSEA=0.026, GFI=0.975, CFI=0.970, IFI=0.968), with item standardized factor loadings ranging from 0.62 to 0.85. The high reliability and validity of the scale ensured accurate measurement of mobile phone dependence levels, providing a rigorous empirical tool for analyzing its relationship with loneliness and physical exercise.

（4）Sport Motivation Scale

This study employed the Exercise Motivation Scale (SMS) developed by Pelletier et al. based on the Theory of Self-Determination. The scale distinguishes three major types: Extrinsic Motivation, Intrinsic Motivation, and Amotivation, comprising seven subscales with 28 items and a five-point Likert scale (Zhang et al., 2023). Extrinsic Motivation involves external rewards or pressures, Intrinsic Motivation focuses on intrinsic interest, achievement, and stimulating experiences, while Amotivation reflects a lack of motivation. The scale demonstrated good reliability and validity through confirmatory factor analysis (χ²/df=2.37, RMSEA=0.028, GFI=0.977, CFI=0.972, IFI=0.971), effectively measuring the multidimensional characteristics of individual exercise motivation. This study utilized the scale to explore the moderating role of exercise motivation in the relationship between physical exercise and mobile phone addiction among college students, providing a robust measurement tool for deeper understanding of the underlying mechanisms and ensuring the scientific rigor of the research.

3.3 Data Collection

In the early stage of the study, the team first reviewed various scales to ensure their reliability and validity. Subsequently, the researchers designed and distributed electronic questionnaires using the China's Wenjuanxing platform, adopting a random sampling method to collect data from college students in China. The questionnaires were disseminated via QR codes and collected over a 30-day period, ultimately yielding 10,676 valid responses. The research team ensured data sufficiency through extensive online dissemination. All questionnaire results were imported into Excel sheets after collection to prepare for subsequent data analysis and processing. This process effectively guaranteed the smooth progress of data collection and laid a solid foundation for subsequent statistical analysis. Samples with more than 15% missing data were excluded, and random missing values in the remaining samples were handled using Multiple Imputation to ensure data integrity.

3.4 Scale Reliability and Validity Analysis

3.4.1 Reliability Analysis

This study analyzed the reliability of the measurement tools using Cronbach's Alpha coefficient to evaluate their validity. SPSS 21.0 software was employed to analyze 68 measurement items. The results demonstrated that the Cronbach's Alpha for the Physical Exercise Scale was 0.797, which, although slightly lower, was acceptable; the Loneliness Characteristics Scale yielded a value of 0.846, indicating high internal consistency; the Smartphone Addiction Scale (SAS) achieved a value of 0.932, demonstrating extremely high reliability; while the Exercise Motivation Scale reached a value of 0.967, showing exceptionally strong reliability and consistency. All scales exhibited Cronbach's Alpha values ≥0.70, ensuring the reliability of the study findings, particularly in the areas of loneliness, smartphone dependence, and exercise motivation, thereby providing a solid data foundation for subsequent research.

***Table 2 Reliability Analysis Overview of Each Scale***

| Scale | Number Of Terms | Cronbach's Alpha |
| --- | --- | --- |
| Physical Exercise Scale | 3 | 0.797 |
| UCLA Loneliness Scale (3rd Edition)） | 20 | 0.846 |
| Smartphone Addiction Scale (SAS) | 17 | 0.932 |
| Sport Motivation Scale | 28 | 0.967 |

This study employed exploratory factor analysis to evaluate the structural validity of the scales, ensuring the stability and consistency of latent variables. Analysis was performed using SPSS 21.0, and the basic data requirements were validated: all KMO values were greater than 0.7, and the Bartlett's test for sphericity was significant. Specifically, the Physical Exercise Scale (PES) had a KMO of 0.730, the UCLA Loneliness Scale (ULS) had a KMO of 0.933, the Smartphone Addiction Scale (SAS) had a KMO of 0.937, and the Exercise Motivation Scale (EMS) had a KMO of 0.980, all demonstrating good structural validity. The KMO values and Bartlett's test results for all scales indicated strong correlations among the observed variables, making them suitable for factor analysis. Therefore, the scales used in this study exhibit high structural consistency and rationality, providing reliable theoretical support for subsequent research.

***Table 3 Summary of KMO and Bartlett's Test for Each Scale***

| Scale | KMO | The sphericity test of the Bartlett | |
| --- | --- | --- | --- |
| Physical Exercise Scale | 0.730 | Approximate chi-square test | 6578.276 |
|  |  | df | 3 |
|  |  | Sig | 0.000 |
| UCLA Loneliness Scale (3rd Edition)） | 0.933 | Approximate chi-square test | 136359.246 |
|  |  | df | 190 |
|  |  | Sig | 0.000 |
| Smartphone Addiction Scale (SAS) | 0.937 | Approximate chi-square test | 117073.597 |
|  |  | df | 136 |
|  |  | Sig | 0.000 |
| Sport Motivation Scale | 0.980 | Approximate chi-square test | 272850.336 |
|  |  | df | 378 |
|  |  | Sig | 0.000 |

Test-retest reliability measures the consistency between two identical assessments of the same group of participants. Calculated as the Pearson product-moment correlation coefficient of scores from two consecutive tests, this metric not only reflects changes in test results but also evaluates score stability. Hence, it is also termed the reliability of repeated measurements or the stability coefficient of measurement. In this study, 50 college students who had completed the initial questionnaire were retested one month later. Pearson correlation analysis was used to assess the correlation between the two test results, aiming to evaluate the degree of consistency between the students' scores before and after the retest. The results demonstrated that all four scales exhibited test-retest correlations exceeding 0.8, indicating strong correlation and consistent data between the two assessments, which provides valuable insights for subsequent research.

***Table 4 Test-Retest Reliability Analysis of the Scales***

| Scale | Number Of Terms | Correlation Coefficient Of Two Tests |
| --- | --- | --- |
| Physical Exercise Scale | 3 | 0.886 |
| Ucla Loneliness Scale (3rd Edition)） | 20 | 0.903 |
| Smartphone Addiction Scale (SAS) | 17 | 0.879 |
| Sports Motivation Scale | 28 | 0.892 |

Psychological network estimation and visualization were performed using the 'qgraph' and 'bootnet' packages in R software (Version 4.3.1).
